# Supplementary material for: Retrospective Cohort Study of the Effectiveness of the Sputnik V and EpiVacCorona Vaccines against the SARS-CoV-2 Delta Variant in Moscow (June–July 2021)
Source: Vaccines (Basel). 2022 Jun 21;10(7):984. doi: 10.3390/vaccines10070984 (PMC9320764; doi:10.3390/vaccines10070984)
Supplement: Supplementary file 1 [file vaccines-10-00984-s001.zip › vaccines-1775389-supplementary.pdf]

Supplemental Table S1. COVID-19 cases in Moscow categorized according to age and vaccination status of patients (June-July 2021).

Age	completed vaccination	month	The outcome and form of COVID-19 infection										in Moscow		
			death		severe form		moderately severe		light form		asymptomatic		vaccin.	seronegative	population
			unvac.	vac.	unvac.	vac.	unvac.	vac.	unvac.	vac.	unvac.	vac.	by the 1st of the month		2021
18-50	Sputnik V	June	158	1	974	26	10463	368	88075	5703	14623	1027	711347		5760116
		July			645	13							879731	2947963	
	EpiVacCorona					7							27529		
51-70	Sputnik V	June			2429	103	10009	833	27525	4726	4202	751	512338		3284276
		July			1819	92							580664	1790209	
	EpiVacCorona					24							17910		
71+	Sputnik V	June			3153	174	5151	606	6221	1701	1398	364	129466		1422425
		July			2418	151							138974	831713	
	EpiVacCorona					30							3206		
50+	Sputnik V	June	1700	70	5582	277	15160	1439	33746	6427	5600	1115	879731		4706701
